# The role of manufacturers in the implementation of global traceability standards in the supply chain to combat vaccine counterfeiting and enhance safety monitoring

**DOI:** 10.1016/j.vaccine.2020.11.011

**Published:** 2020-12-14

**Authors:** Stephen Jarrett, Taufik Wilmansyah, Yudha Bramanti, Hikmat Alitamsar, Drajat Alamsyah, Komarapuram R. Krishnamurthy, Lingjiang Yang, Sonia Pagliusi

**Affiliations:** a Gracious International Inc., 28 Jiafeng Road, Shanghai 200131, China; b PT Bio Farma (Persero), Jl. Pasteur No. 28, Bandung Jawa Barat 40161, Indonesia; c Bharat Biotech, Genome Valley Shameerpet, Hyderabad 500078, Telagana, India; d ChengDu Institute of Biological Products Ltd., 379 3 Section Jinhua Road, Jinjiang District, Chengdu 610023, China; e DCVMN International, Route de Crassier 7, 1262 Eysins-Nyon, Switzerland

**Keywords:** Vaccine coverage, Supply chain, Traceability, Barcode, Counterfeit, Safety monitoring

## Abstract

The counterfeiting of vaccines is an increasing problem globally with the safety of persons vaccinated, the trust in vaccines generally and the associated reputation of vaccine manufacturers and regulatory agencies at risk. This risk is especially critical with the on-going development of COVID-19 vaccines. The ability to track and trace vaccines through the vaccine supply chain down to persons vaccinated has to be enhanced. In this context of traceability, the global immunization community has recently set the barcoding of the primary packaging of vaccines, specifically vaccine vials and pre-filled syringes, as a top priority. Emerging vaccine manufacturers are already engaged in investigating ways to incorporate barcoding in their labelling and packaging using GS1 international standards. A specific pilot taking place in Indonesia by the national vaccine manufacturer, Bio Farma, shows the innovation of barcoding on primary packaging already underway with a relatively modest level of investment and success at this stage. This article highlights the efforts of industry and governments on the value of traceability and introduction to 2D barcodes. Access to financial resources and support from the international immunization community would accelerate such innovations leading to enhanced security of the vaccine supply chain.

## 1 Introduction and background

The distribution of sub-standard, defective, expired or counterfeit vaccines is a threat to the health of diverse populations around the world. The International Institute of Research Against Counterfeit Medicines (IRACM), an independent international organization created a decade ago, has already recorded numerous instances of counterfeit vaccines principally in Africa and Asia (Table 1). As one example, the World Health Organization (WHO) issued a medical alert in 2019 about falsified rabies vaccines circulating in the Philippines, including falsified versions of products from multinationals and from one company in China [1]. In 2018, China experienced a public health crisis with the administration of over 600,000 sub-standard DTP vaccines in children [2]. These counterfeit products present a risk for the safety of the public.

**Table 1:** Recorded events of countries where counterfeit vaccines were identified.

Year	Vaccine type	Country where counterfeit vaccines were identified
2020	COVID-19	Russia
2020	COVID-19	Ecuador
2019	Rabies	Philippines
2019	HPV	Hong Kong
2019	Meningitis	Cameroon
2019	Cholera	Bangladesh
2019	Meningitis	Niger
2018	Chickenpox	Venezuela
2018	Hepatitis B	Uganda
2017	DTP	China
2017	Meningitis	Nigeria
2016	Rabies, meningitis	China
2016	Polio, hepatitis B	Indonesia
2016	Yellow fever	Angola
2016	Tetanus toxoid	Philippines
2015	Meningitis	Niger

Illustrative reports resulting from a search, using the term vaccine, on the International Institute of Research Against Counterfeit Medicines, covering some posts since 2015. Cf. http://www.iracm.com/en/?s=vaccine&submit=Search, accessed 23 September 2020.

The WHO global surveillance and monitoring system uses the terminology of substandard and falsified medical products [3]. For this paper, however, the term counterfeit is used throughout to include sub-standard, falsified and fake products.

While events of counterfeit vaccines seem to be increasing in recent years, not all these events are identified and recorded, triggering an increased focus on this issue. Not only are counterfeit products highly profitable but the internet has made sales and the trade of such products simpler and more global^1^. Counterfeit is identified usually through monitoring carried out by the Ministry of Health and its personnel, through spot checks, especially in private outlets. In terms of the value of all pharmaceutical products seized by customs globally between 2014 and 2016, more than 80% originated in Asia, e.g. China and India [4]. Both countries have taken action intended to reduce the volume of counterfeit products, and monitoring vaccines’ supply over time will provide evidence as to whether these actions have been successful in fighting counterfeit. The negative health consequences of counterfeit vaccines’ supply is mainly failure to immunize subjects, causing the potential for catching the disease with the ensuing loss of confidence in vaccines and credibility of vaccine manufacturers in general.

China established a new Law on Vaccine Administration which took effect on 1 December 2019, with regulatory requirements for researching, producing, distributing and using vaccines, and setting up a national vaccine electronic tracking platform [5]. The Law, comprising 11 chapters and 100 articles, covers vaccines in the national immunization schedule and for emergencies and other vaccines voluntarily used by the public. The market authorization holder, which has to have a manufacturing license, is responsible for the safety, efficacy and quality control of each vaccine. Specific penalties for violations, which are reportedly stricter than other drug laws, are stipulated, along with a compensation system for abnormal adverse events.

India has had since 2011 a track and trace system incorporating barcode technology as per GS1 international standards^2^ for all pharmaceutical products exported from the country, with barcoding at secondary and tertiary packaging levels mandatory but still optional at the primary packaging level [6]. In 2019, the Government made it mandatory for all medicines procured under Public Procurement from 1st April to exhibit or display barcoding at primary packaging level [7], although vaccines are not specifically mentioned in this notification.

Counterfeiting of vaccines undermines the reputation of and trust in both manufacturers and regulators. It is a real threat particularly during the current COVID-19 pandemic. The US Food and Drug Administration (FDA) has already alerted the public to the possibility of fraudulent COVID-19 vaccines [8]. Interpol has already seen a rise in fake medical products related to COVID-19 [9]. One warning even indicates the potential for a parallel crisis of substandard and falsified products [10]. A counterfeit and potentially dangerous coronavirus vaccine is reportedly already being sold in South America.^3^

Given the large amounts of resources being invested in the research and development of COVID-19 vaccines, the likelihood of counterfeit vaccines reaching global markets is very high. As the country where the coronavirus outbreak began, China was fast in developing vaccines. Examples include Sinopharm, a state-owned pharmaceutical company based in Beijing, which is developing two vaccines made using parts of the coronavirus that have been inactivated, so that they can no longer cause disease, and both vaccines had been reported to produce antibodies in all participants in preliminary phase I and II trials. In addition, Beijing-based company Sinovac has announced similarly promising results for its own inactivated-virus vaccine, under a phase III trial in Brazil,^4^ while Sinopharm announced testing its inactivated vaccines in the United Arab Emirates (UAE).^5^ CanSino Biological Inc. with the Beijing Institute of Biotechnology also have a vaccine in a phase III trial. In India, Bharat Biotech, located in Hyderabad, has an inactivated-virus vaccine that has shown promise in phase I trials and has been approved for a phase II trial.^6^ Zydus Cadila located in Ahmedabad has also been approved for a phase II trial for its DNA vaccine.^7^

While national regulatory authorities have the task of assuring the proper registration and licensing of vaccines for human use, counterfeit products can easily bypass their scrutiny due to complex and fragmented supply chains. Distributors and consumers, including for example privately-owned health care systems, faced with multiple product options can inadvertently receive and use counterfeit products, especially if prices are attractive.

One of the solutions to confronting counterfeiting is to ensure the unique identification of registered and licensed vaccines and then to be able to track their path from manufacturer through to the individual being vaccinated. The Developing Countries Vaccine Manufacturers Network (DCVMN), comprising 41 manufacturers from emerging countries in Africa, Asia and the Americas, has put the challenge of track and trace, or traceability, as one of its priorities in supply chain efficiency [11]. Additionally, the Network has already indicated the importance of traceability in the context of COVID-19 vaccines where Network members are developing over 20 candidate vaccines [12].

Traceability is the capability of tracking vaccines with key data such as the Global Trade Item Number (GTIN), used by a company to unambiguously identify all of its trade items. The GTIN is a standardized and registered numbered code that incorporates manufacturer, lot number and expiry date [13]. With appropriate systems in place in emerging countries these data support inventory management, consumption history and coverage rates, wastage and demand forecasting. Such data are crucial for vaccine safety monitoring through pharmacovigilance, detecting, assessing, understanding, preventing and reporting adverse events.

Full traceability of vaccines, from factory to individuals receiving each dose, will increasingly be demanded from countries around the world implementing digital health systems (e.g. eHealth/mHealth) for improving their immunization information systems and monitoring vaccine coverage rates. At least 120 countries are engaged in implementing or developing such digital health systems [14]. Vaccine manufacturers will, therefore, have to adopt full traceability measures to meet this growing demand to retain or expand their vaccine market shares.

**Fig. 1:**
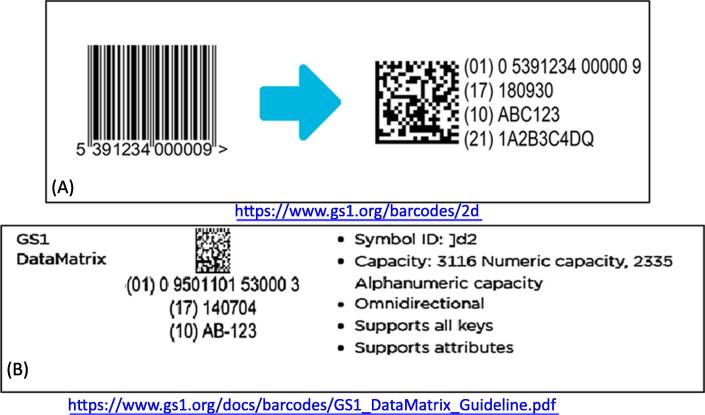
Illustration of the GS1 2D DataMatrix barcode. (A) This is an illustration of GS1 barcode and corresponding DataMatrix, which is a 2D matrix (or two-dimensional) barcode which may be printed as a square or rectangular symbol made up of individual dots or squares [16]. The 2D representation is an ordered grid of dark and light dots bordered by a finder pattern. The finder pattern is partly used to specify the orientation and structure of the symbol. The data is encoded using a series of dark or light dots based upon a pre-determined size. The size of these dots is known as the X-dimension. (B) A single 2D barcode can hold a significant amount of information and may remain legible even when printed at a small size or etched onto a product. 2D barcodes are used in a wide range of industries, from manufacturing and warehousing to logistics and healthcare.

Barcoding is a principal tool used in traceability as it encompasses the numbered code which is rapidly read by laser or photographic tools. Bar codes are recommended by WHO on all vaccine packaging levels used by manufacturers, with the exception of primary packaging, and should conform to GS1 standards [15]. New two-dimensional (2D) barcodes look like squares or rectangles that contain a series of many small, individual bar codes superposed within the square space. A single 2D barcode can hold a significant amount of information and may remain legible even when printed at a small size or etched onto a product; barcode size between 5 mm and 10 mm square has been shown to be successfully scanned taking account of vial curvature (Fig. 1) [16].

An important development is the recent decision of the Vaccine Innovation Prioritisation Strategy (VIPS) Alliance of WHO, Gavi, the Bill & Melinda Gates Foundation, the United Nations Children's Fund (UNICEF) and PATH to prioritize the development and implementation of barcoding on primary packaging [17]. This decision has come after three years of collaboration, reviewing vaccine product innovations aimed at simplifying logistics and increasing the safety of immunization. Next steps will include the mobilization of resources, the advancing of innovation and the development of appropriate policies.

The importance of barcoding on primary packaging relates to a much easier and quicker method of retrieving data, in anticipation of countries' increasing adoption of digital health information systems. Barcoding also has the ability to add more data, including serialization, at a future stage.

## 2 Implementing GS1 traceability standards

To date, 17 vaccine manufacturers which are DCVMN members, have the capability to apply barcoding on secondary and tertiary packaging using GS1 standards. As an example, Bharat Biotech in India is currently organizing barcoding using these standards, following the national directive mentioned above to address counterfeit products and ineffective recall challenges in the country. The process being followed includes the following steps:

1. Engaging with the GS1 Organization to secure a valid GS1 company prefix
2. Training and educating staff members responsible for labelling and packaging through standard operating procedures (SOPs) on the implementation of GS1
3. Allocating a globally unique GTIN for each product item, with the company prefix, item reference number, check digit and an indicator digit where 0 is for primary packaging, 1 for the innermost level of secondary packaging, 2 for the second level of secondary packaging, 4 for the outermost level of secondary packaging, and 5 for tertiary packaging.
4. Linking GTINs in the internal software applications, capturing the GTIN and other related attributes like name, description and content, ensuring GTIN uniqueness for new products to avoid duplication and building an automatic check digit calculator into the system.
5. Implementing and validating track and trace equipment, preparing the user requirement specifications, procuring equipment, initiating control changes, installing and qualifying equipment.

The below case study from Bio Farma Indonesia, an emerging country vaccine manufacturer, indicates that using standard GS1 2D barcoding directly on vaccine vials is both viable and achievable at relatively low capital cost. In fact, 2D barcoding is already on primary packaging, including vials and pre-filled syringes, for vaccines produced by some multinational companies for use in developed/industrialized countries [18].

## 3 Case Study: Piloting traceability innovation by PT Bio Farma (Persero) Indonesia

Problem Statement:

Bio Farma, formed in 1890, is Indonesia's state-owned vaccine manufacturer under the Ministry of State-Owned Enterprises, focusing on research, development, production, marketing and distribution of biological and pharmaceutical products both nationally and globally. It plays an active role in conducting research on new vaccines to ensure a sustainable vaccine supply in Indonesia, as well as meeting global demand for high quality and affordable vaccines. Since 1997, Bio Farma has achieved WHO pre-qualification for 16 vaccine presentations related to 7 types of vaccine-preventable diseases including poliomyelitis, diphtheria, tetanus, whooping cough, *Haemophilus influenzae*, measles and hepatitis B.^8^

Indonesia has experienced a national issue regarding the counterfeiting of vaccines by non-registered manufacturers found on the local market, impacting the products of Bio Farma. In May 2016, in Bekasi close to Jakarta hundreds of fake vaccines were seized, including products sold as polio and hepatitis B treatments. In the case of Indonesia, the legal consequences for vaccine counterfeiters was prison for the perpetrators; those arrested confessed to selling such vaccines throughout Indonesia since 2003 in around thirty hospitals and community health centres [19].

**Fig. 2:**
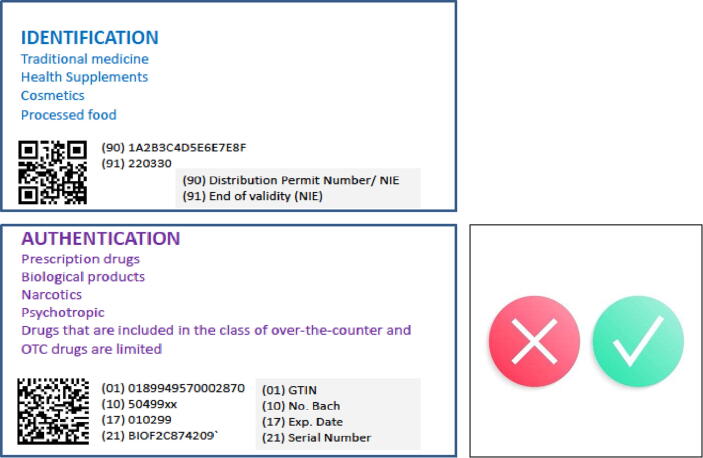
Differential Use of QR code and Matrix code in the Indonesian track and trace system Traceability demands that codes be readable from the beginning until the end of their lifecycle. Both data matrix codes and QR codes are scalable, but small components such as electronic devices are typically marked with data matrix codes since they can encode more characters within the same space. Some markings have cells that are as small as 300 μm^2^, whereas other markings are as large as a 1 m2. QR codes are less compact in size and are therefore not typically used for small items. In Indonesia the regulatory authority defined that 2D data Matrix is to be used for biologicals including vaccines, and other prescription drugs, instead of QR. Upon scanning with a smartphone the screen will show specific information and whether the product could be used or not, based on recognition from the integrated data system.

Subsequently, the National Agency of Drug and Food Control (Badan POM or BPOM) issued a regulation, Number 33 of 2018, requiring the implementation of 2D barcodes in the control of drugs and food [20]. Importantly, the regulation distinguishes between the use of the 2D QR barcode for traditional medicine, health supplements, cosmetics and processed food and the higher-capacity 2D Data Matrix barcode for prescription drugs, biological products, narcotics, psychotropic drugs and limited over-the-counter drugs (Fig. 2).

Bio Farma has addressed the vaccine counterfeit issue by an innovation implementing the full traceability of their vaccines down to the person vaccinated, allowing for validating the authentication of all of Bio Farma's vaccines used in vaccination programmes and, importantly, providing safety for every person vaccinated.

### 3.1 Business decision-making

The decision to implement this innovation in traceability, approved by the Bio Farma management, was based on references available both globally and from WHO regarding problems with the counterfeiting of vaccines that Bio Farma was experiencing. The specific benefits expected include increased product security from counterfeiting, prevention of diversion and theft and patient safety through product barcoding with unique identification and authentication systems.

The innovation focused on process integrity and transaction security at the sales unit level, providing a chain of custody and accountability from manufacturer to persons vaccinated. This protected the business reputation of Bio Farma and enhanced trust in vaccines, providing full supply chain visibility, combatting reimbursement fraud and ensuring recall effectiveness if needed.

Complete digitization through an 'e-supply-chain' was considered both transformational and sustainable, with credible and verifiable sales unit level data facilitated by the use of mobile computing.

The investment for this innovation has been allocated over a two-year period divided into two main components:

• Hardware: Modification and improvement of the existing packaging line consisting of additional hardware (compatible camera sensor, 2D code printing system, mechanical adjustment);
• Software: Development of a track and trace system from labelling through to distribution, with a mobile track and trace application from manufacturer through to end user.

**Fig. 3:**
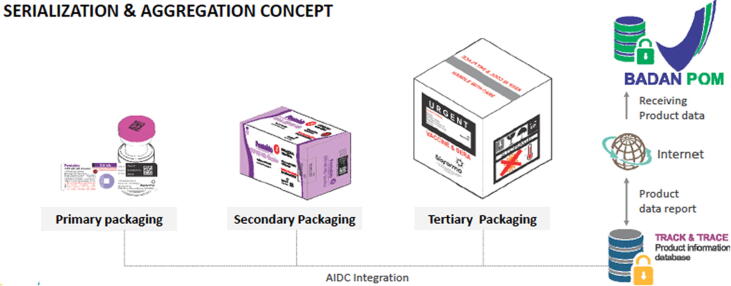
Automatic Identification and Data Capture (AIDC) flow from primary to tertiary packaging level. Automatic Identification and Data Capture (AIDC) refers to the methods of automatically identifying objects, collecting data about them, and entering that data directly into computer systems, without human involvement, i.e. barcodes of standards on secondary and tertiary packaging levels for vaccines. They facilitate product and data flow between supply chain partners.

The principal expenditure was the installation of the integrated line for labelling and cartoning, which allowed for a continuous and seamless adoption of unique identifiers on primary, secondary and tertiary packaging, as illustrated in Fig. 3.

### 3.2 Target innovation profile

The principal goal of the innovation was to secure ease of tracking vaccines down to the smallest distributor and on to the person being vaccinated. The main target was to apply vaccine data to primary packaging (vaccine vials) specific to each product with the GTIN, serial number, batch number and expiry date. The specific data in every primary packaging level (vials) will be aggregated using indicator digits for secondary packaging levels (packs with several vials) and the same for tertiary packaging (cartons with several packs). Central to the profile was ensuring full compatibility with the use of smart phones for data download given all health workers in Indonesia are equipped with smart phones. This data management system enables authentication of the product, preventing other parties from counterfeiting the products.

Global supply requirements, as recommended by WHO, guided the overall profile, and Bio Farma registered for using GS1 standards as shown above. The 2D Data Matrix barcode was used as data carrier, which is standard for many health care products allowing a large amount of data to be included in a small space with variable information at high production rates. Bio Farma generates its own barcode.

### 3.3 Innovation design

The design strategy was based on research into technology standards and availability, national and international market requirements and ways to eliminate product counterfeiting. This included looking at global trends in traceability and discussions with several related parties such as the pharmaceutical industry, national and international regulatory authorities and hardware and software suppliers, among others.

The focus was on a new system of packaging, with implementation consisting of several phases, the first being the distribution of innovative packaging to the government sector in Indonesia as a pilot project before regional and national roll-out. Bio Farma itself designed the innovation in consultation with the BPOM and with support in utilities and technology from international companies. A key challenge in the design was related to the printing area required for the 2D Data Matrix barcode on the label, along with other required information and a vaccine vial monitor (VVM).

Several factors were taken into account in determining the attributes of the barcode, including the effect of size, vial curvature, print fading and ambient light, to maximize scan success rates using smart phones, noting previous trials in this regard [21].

The success of this innovative track and trace system was dependent on the involvement of all relevant stakeholders including health workers in the field to ensure they were able to implement the protocol adopted. Broad social mobilization was carried out throughout all parties concerned about the importance of implementing this system. The ease of use, either through a scanner or the use of a smart phone created an open, attractive and time-saving information system.

A total of 1200, 5-dose vaccine vials used in the design have been tested and evaluated in one provincial level distribution area, supplying 6000 vaccine doses to three districts/city catchment areas. Public health facilities and vaccinated persons participated in the pilot testing phase.

### 3.4 Process design

**Fig. 4:**
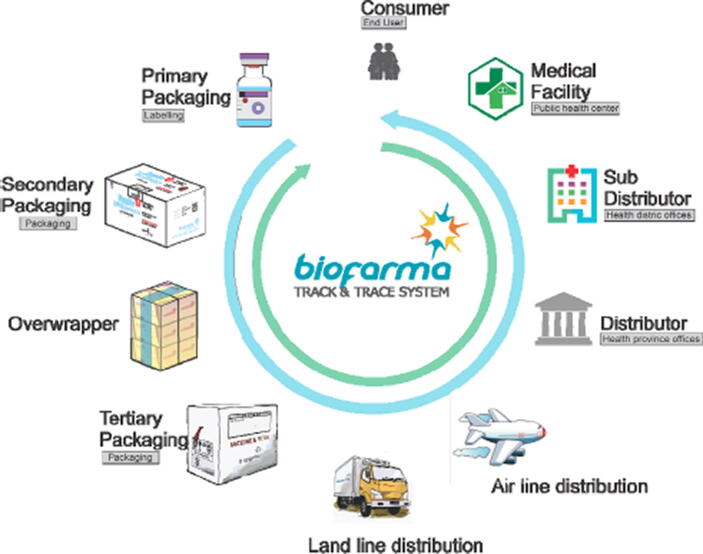
Vaccine traceability in Indonesia from primary packaging to consumer BioTracking, from primary packaging to consumer (following the blue arrow in Fig. 4), is a logistics system for vaccines requiring login access and used by distribution staff receiving vaccines from the manufacturer to the point of vaccination to verify and authenticate the product received, logging the receipt and release of products from each distribution unit. BioDetect, which does not require login access, is a downloadable mobile application used by the general public to enable verification of the authenticity of the product and the status of product use at time of vaccination, allowing feedback from the consumer to point of manufacturer (following the green arrow in Fig. 4).

Given the unique situation of Bio Farma as a state-owned enterprise, the innovation included not only adaptation during the manufacturing process but also the design of software and an application enabling implementation in the whole territory of Indonesia. As such, the innovation covers packaging (labelling, cartoning, quarantine and release), as well as distribution channels, delivery points and vaccine use (Fig. 4).

This innovation in traceability comprising both product movement and use consists of two software applications – BioTracking and BioDetect – both of which were developed internally by Bio Farma.

Bio Farma developed its own customized software programme and application, using a Microsoft MySQL server. This ties into the mHealth implementation strategy under the Ministry of Health and BPOM regulations. Data from persons vaccinated will be posted in the application and integrated into the immunization information database.

### 3.5 Process performance

At the manufacturing level, the track and trace system relates to the adaptation of the packaging line, situating the barcoding on primary, secondary and tertiary packaging. Central to this were the additional elements of a 2D barcode printing system, a compatible camera sensor and mechanical adjustments to the line. A smart printer is used for the 2D barcode print and its system can be integrated to the track and trace software through an interface. The camera can read the 2D barcode reliably at production speed and can also be integrated to the track and trace software through an interface.

For quality control, each stage of the process is sampled and then manually verified as to the suitability of the data and the reliability of the system. Data validation ensures there are no untraceable products or incapability to track data down to persons being vaccinated. A validation report documents issues relating to installation quality (IQ), operational quality (OQ) and performance quality (PQ).

Standard operating procedures (SOPs) were developed for 5 distinct processes; labelling, cartoning, manual packing, warehousing, applications use.

### 3.6 Regulatory oversight

National regulators in BPOM were consulted in the innovation design. Standing regulations in Indonesia require pharmaceutical products to be labelled with 2D barcodes for authentication. This traceability innovation incorporates vaccines into these standing regulations, being also in line with WHO requirements for labelling packaging with a standard GS1 barcode. Regulatory oversight monitors the implementation of the packaging including the registration, appropriateness and reliability of the codes generated.

### 3.7 Human resources

Around 20 staff were involved in the development and management of the BioTracking and BioDetect traceability system, principally from the packaging, information technology and quality assurance sectors. This same team has been involved in training Bio Farma staff in the track and trace system, regulatory requirements and the technical aspects of implementation. They have also been involved in training the relevant staff at provincial and local levels in the pilot phase of the project. As yet, there is no detailed plan for the training of health staff in the roll-out and nationwide implementation of the system.

### 3.8 Investment

Adaptation of the vaccine packaging line has been the principal cost in terms of hardware, amounting to between the equivalent of US$1 to 2 million. Software development and testing may run at about a similar level of investment. As it is still in a pilot stage, the full and longer-term costs will become clearer once the pilot phase is completed.

For manufacturers generally, investment, in addition to adopting GS1 standards, will have to be clearly delineated between adapting packaging line(s) with the appropriate software and quality control actions required and the broader traceability systems undertaken nationally where these are developed separately by Ministries of Health. If the barcoding innovation is implemented only at the manufacturer, modifying the labelling and packaging line, the overall cost is more modest as software development is not extensive. The level of investment may vary depending on whether manufacturers are already registered with GS1 and whether they already have a GTIN registry, although hardware and software costs would still have to be covered.

## 4 Discussion and lessons learnt

The two principal drivers influencing innovation in traceability are regulatory requirements and the specific needs of markets that manufacturers supply with vaccines. Both need to be carefully assessed and well understood. Where manufacturers supply vaccines to national markets, traceability innovation has to be compatible with the national immunization information system.

The adoption of GS1 traceability standards, as recommended by WHO and required by Gavi, is now recognized across the vaccine industry and all manufacturers will need to comply in order for their vaccines to be accepted internationally, especially when procured by UNICEF and also certainly for European and North American markets.

A principal challenge was to integrate existing systems that had not been integrated before, especially as the system in Indonesia involves the complete immunization information system, with traceability from manufacturer down to persons vaccinated. A key component for success was communication with all partners who are instrumental in successful implementation including authorized distributors, private health care facilities and health workers in the field.

At Bio Farma, the main challenge was not on label modification, but on modifying existing equipment and software systems. One specific challenge was the fact that not all vials are steady in one position in the line complicating the reading and verification of labels. Manual adjustment in the first step of process on the line between label verification and labelling is used to ensure vials are able to be accurately read. The ability to read the labels to verify data is essential to ensure correct matching with labels on cartons for shipment.

The intervention has focused on vials. As yet, the accuracy of reading barcodes compared to earlier manual recording has not been assessed, nor whether this is consistent with similar assessments that have been carried out elsewhere [22]. Ampoule and Uniject™ labels have not been tested; both may present challenges because the label area is very small and adding 2D barcodes may not be feasible. Whether additional primary packaging, or an extended label, around each unit would be needed in order to add a 2D barcode needs to be investigated, recognizing that there could be additional challenges in label data verification and packaging.

The Bio Farma innovation developed over a two-year period as it covered actions both at the manufacturer and in the country-wide immunization information system. Implementing this barcoding innovation only at the manufacturer site, including the adoption of GS1 standards, would entail a short time period of between 12 and 18 months.

## 5 Conclusion

With the risk of counterfeiting and the increasing use by countries of digitization of their health information systems, the placement of 2D barcodes on primary packaging is rapidly evolving as an essential standard requirement for vaccines globally. This practice offers the additional benefit of tracking the dose administered to each person vaccinated and allows for accruing data on vaccine coverage at country level.

The imminent global distribution and use of potential COVID vaccines in large emerging countries warrants the use of global traceability standards, instead of local traceability systems, which could tremendously facilitate the monitoring of post-approval studies (or phase IV studies), tracking vaccines received by each subject worldwide, that will likely be necessary to determine the effectiveness of vaccines developed in an emergency context and record timelines. This will accelerate accruing data on safety and effectiveness in communities and populations in the field.“

The Bio Farma pilot case study indicates that implementation of GS1 traceability standards to the level of vaccine primary packaging is feasible, while requiring modest investments. This can bring benefits to subjects receiving the vaccines based on different batch numbers and manufacturing facilities. In addition, governments and public health agencies seeking accurate data on vaccine distribution and coverage, as well as safety monitoring and effectiveness, are likely to benefit most from the implementation of global traceability standards. The benefits will likely outperform the investments, but this was not addressed in this pilot, as it requires a long term followup.

DCVMN will continue prioritizing the issue of vaccine traceability, both the adoption of GS1 standards and the barcoding of primary packaging, given the priority recently given to this by the global immunization community, but also to ensure the widespread safety of vaccines. The fact that one DCVMN member is already well advanced in adding 2D barcodes to primary packaging is an important step forward. Its experience will be shared as a best practice for other manufacturers to emulate, helping counter the spread of counterfeit vaccines and ensuring the public retains trust in vaccines and vaccination while, at the same time, upholding manufacturers' reputation. This case study report highlights the joint efforts of industry and governments on the value of traceability and introduction to 2D barcodes.

DCVMN calls on the global immunization community to consider supporting, potentially on a cost-share basis, emerging vaccine manufacturers who voluntarily decide to implement traceability systems that allow for tracking vaccines down to persons vaccinated, contributing to vaccine safety and helping countries effectively manage their vaccine supply chain and enhance their immunization information systems.

## 6 Disclaimer

The authors alone are responsible for the views expressed in this article, which do not necessarily represent the views, decisions or policies of any mentioned institutions with which the authors are affiliated.

## Declaration of Competing Interest

The authors declare that they have no known competing financial interests or personal relationships that could have appeared to influence the work reported in this paper.

## Footnotes

1 https://apnews.com/ef15478fa38649b5ba29b434c8e87c94 accessed 19 September 2020.

2 https://www.gs1.org/standards

3 https://www.jpost.com/health-science/fake-coronavirus-vaccine-with-hebrew-label-being-sold-in-south-america-629416

4 https://www.businesswire.com/news/home/20200706005305/en/Sinovac-COVID-19-Vaccine-Collaboration-Butantan-Receives-Approval

5 https://www.reuters.com/article/us-health-coronavirus-emirates-vaccine/chinas-sinopharm-begins-late-stage-trial-of-covid-19-vaccine-in-abu-dhabi-idUSKCN24H14T

6 https://www.clinicaltrialsarena.com/news/bharat-biotech-covaxin-data/

7 https://www.clinicaltrialsarena.com/news/zydus-covid-vaccine-phaseii/

8 https://extranet.who.int/pqvdata/, accessed 10 August 2020.
